# Frequency Disentanglement Distillation Image Deblurring Network

**DOI:** 10.3390/s21144702

**Published:** 2021-07-09

**Authors:** Yiming Liu, Jianping Guo, Sen Yang, Ting Liu, Hualing Zhou, Mengzi Liang, Xi Li, Dahong Xu

**Affiliations:** 1 College of Information Science and Engineering, Hunan Normal University, Changsha 410081, China; liuyiming@hunnu.edu.cn (Y.L.); 202070291671@hunnu.edu.cn (S.Y.); tinaliuting2021@163.com (T.L.); 202020291657@hunnu.edu.cn (H.Z.); 202020291651@hunnu.edu.cn (M.L.); lixi@hunnu.edu.cn (X.L.); xudahong@hunnu.edu.cn (D.X.); 2 College of Physical Education, Hunan Normal University, Changsha 410081, China

**Keywords:** image deblurring, feature disentanglement, distillation block, frequency split

## Abstract

Due to the blur information and content information entanglement in the blind deblurring task, it is very challenging to directly recover the sharp latent image from the blurred image. Considering that in the high-dimensional feature map, blur information mainly exists in the low-frequency region, and content information exists in the high-frequency region. In this paper, we propose a encoder–decoder model to realize disentanglement from the perspective of frequency, and we named it as frequency disentanglement distillation image deblurring network (FDDN). First, we modified the traditional distillation block by embedding the frequency split block (FSB) in the distillation block to separate the low-frequency and high-frequency region. Second, the modified distillation block, we named frequency distillation block (FDB), can recursively distill the low-frequency feature to disentangle the blurry information from the content information, so as to improve the restored image quality. Furthermore, to reduce the complexity of the network and ensure the high-dimension of the feature map, the frequency distillation block (FDB) is placed on the end of encoder to edit the feature map on the latent space. Quantitative and qualitative experimental evaluations indicate that the FDDN can remove the blur effect and improve the image quality of actual and simulated images.

## 1. Introduction

Image blur, caused by camera shake, object motion or out-of-focus, is one of the most common visual artifacts. The purpose of image deblurring is to recover a sharp latent image from a blurred image using edge structure and details when a single blurred image is given. Image deblurring has long been an essential task in computer vision and image processing. The image blurring can be expressed by Equation (1). Blurred image, blur kernel, sharp image, noise and the operation of convolution are represented by *B*, *K*, *I*, *N* and ∗ respectively.

(1) B=K∗I+N,

According to whether the blur kernel is a known condition, the deblurring task can be divided into two major branches: non-blind image deblurring [1] and blind image deblurring [2,3,4,5,6,7,8,9,10]. For non-blind deblurring, the information of the blur kernel K must be known in advance. According to the blur kernel, the sharp image can be recovered by reverse convolution on the blurred image. This process is relatively easy to follow by a calculation standard. However, in most cases, the blur kernel is unpredictable. The blind image deblurring is to estimate a sharp image when only the blurred image is given. Obviously, It can be seen that non-blind deblurring is an ill-posed problem. The traditional methods [11,12,13] tend to estimate the blur kernel and the sharp image simutaneously. These methods generally use assumed prior knowledge to limit the uncertainty of the blur kernels, thus turning the blind deblurring problem into a non-blind problem. For example, Ref. [11] believes blur will cause the gradient change of the image. The sharp image is restored by updating the gradient. Pan et al. [13] proposed a dark channel prior constraining method, which can solve this ill-posed problem. However, due to the complexity of blur kernel in the real world, the assumed prior knowledge is inevitably limited and difficult to fully express the blurry situation in the real scene, which leads to the artifacts in the restored image. Moreover, these methods based on iterative optimization techniques are very computationally expensive and usually require tuning a large number of parameters.

With the development of deep learning, many methods [2,3,4,5,6,7,8,9,10] began to no longer predict blur kernels but directly restored sharp images end-to-end by constructing an encoder–decoder structure. Encoder–decoder architecture aims to invert a given blur image back into the latent space by the encoder, and the image then can be faithfully reconstructed from the latent feature by the decoder. Nah [2] has built three parallel encoder–decoder paths, and different paths receive different image scales, so that a sharp image can be gradually recovered from the blurred image. SRNnet [3] also uses a similar multi-scale framework, but it introduced the LSTM [14] to share the intermediate layer information in the latent space. Deblurgan [5] uses the unet-based network as the backbone of the generator. Deblurganv2 [9] replaced unet-based with pyramid network to achieve the effect of feature reuse. Through our observation, although the above method solves the problem of image blurring to some extent. They still cannot restore the original information of the image well and even introduce artifacts inevitably. Through our analysis, such defects are mainly due to the design of the encoder–decoder architecture can not disentangle the blur information from the content information. The encoder task is to extract semantic content feature map from the image, which will served as important clues to reconstruct a high-fidelity sharp image. Then, the decoder is guided by the loss function to supplement the detailed information lost due to the down-sampling in the decoder. It seems reasonable that since the original blur information has also been eliminated mainly in the encoding process. There may be only the content information of the image left in the intermediate feature maps of the latent space. In fact, the blur information of the image is entangled with the content information. Even if the encoder extracts the semantic feature of content information to the maximum extent, there will still be some surviving blur information that is entangled with it so that it is impossible to remove blur features as a single vector or a independent feature channel from the whole feature maps through linear reorganization. These entangled blur features are mistaken as valuable clues in the process of decoder, which disturbs the image reconstruction process of the model and leads to produce unnatural textures and artifacts.

In response to the above problems, we proposed a frequency disentanglement distillation image deblurring network (FDDN) edit on intermediate feature map in the latent space. We proposed the frequency split block(FSB), inspired by octconv [15], disentangles the intermediate feature map in latent space in the dimension of frequency. We think that blur information usually exists in low-frequency features, and semantic feature of content information usually exists in high-frequency features. Extracting blur information from blur image or disentangling blur information from content information is a complicated thing. We cannot solve it directly either, but we can narrow down the scope of solution through our frequency distillation block (FDB). FDB will greatly retain the high-frequency features in the feature channel and distillate the low-frequency features so as to solve the entanglement problem of blur information as far as possible.

There are three clear positive impacts of the FDDN algorithm. First, FDDN is the first time to define the deblurring task as the disentanglement operation of deblurring information and image content information, which is different from the previous encoder–decoder algorithm to generate sharp image directly. Compared with the direct method, FDDN is more instructive to the network and purposefully guides the network to eliminate blurry information, rather than relying solely on the training set and loss function. Second, FDDN algorithm has a great positive impacts in network parameters and running speed, which is due to the FDB disentanglement work at latent space, where the complexity of eigenvectors is the lowest. Third, FDDN has achieved a excellent result both in quantitative and qualitative aspects.

Our contribution can be summarized in the following three points:

- A frequency split block (FSB) is proposed, distilling high-frequency and low-frequency features in different channels.
- We propose a frequency distillation block (FDB) that can better retain the information of the high-frequency characteristic channel and filter and reorganize the information of the low-frequency characteristic channel.
- A lot of experiments have been conducted to prove the validity of the FDDN that we designed.

## 2. Related Work

Image deblurring has also been rapidly developed because of the multi-scale mechanism [2,3,9,16,17]. Nah et al. [2] creatively proposed the multi-scale deblurring pipline at the first time, which introduced three kinds of blurry images with different sizes into the model, and achieve a state of the art result in the year of 2017. This multi-scale design make the model can perceive both detail and semantic information. However, this method required the network to carry out feature extraction and image reconstruction for three times, which lead to the number of network parameters is too large, and the model performance is low. SRN [3] optimized the pipeline of multi scale by employing LSTM mechanism. This design let model share the feature extraction results across scales. However, the problem of parameter overload cannot be solved fundamentally. Zhang et al. [16] investigate a new scheme which exploits the deblurring cues at different scales via a hierarchical multi-patch model, and propose a simple yet effective multi-level CNNs model called Deep Multi-Patch Hierarchical Network (DMPHN), which uses multi-patch hierarchy as input. Gao et al. [17] believed that [2,3] were in two extremes, in which the information of Deep deblurring net [2] was utterly independent at each scale, while SRN net [3] fused all intermediate information without screening and both of them could only obtain suboptimal results. Therefore, [17] proposed a selective sharing mechanism on the basis of multi-scales and solved the complex problem of very deep network training through jump connection. Deblurganv2 [9] also introduces a FPN network [18] into the generator to take advantage of multi-scale feature information, enabling the integration of high-level semantic information with low-level detail information. However, this design still has some shortcomings. The upper semantic information will be diluted in the process of transmission, so the higher semantic information will be gradually weakened in the process of network transmission. Kupyn et al. [5] creatively introduced the model of adversarial generation network in image deblurring, which define the task of deblurring as a transformer task. The generator received blur images as input and sharp images as output, and the discriminator was used to discriminate the authenticity of the generated images.

In 2021, image deblurring [19,20,21,22,23,24] also achieved good results. GCResNet [19] proposed a new codec network, in order to increase the amount of convolution of the graph, the feature map is converted into the vertices of the pre-generated graph to synthesize the structure data of the graph. By doing this, we apply Tulaplacian regularization to feature maps to make them more structured. Pan et al. cited 34 to divide the deblurring process into two steps and proposed a two-stage network. In the first stage, a public convolutional network is used to generate an initial deblurred image. In the second stage, the initial data distribution is transformed into a potential sharp image distribution, and sharp edges are obtained through a priori network. In addition, they proposed a relativistic training strategy aimed at learning the priors of potentially sharp images to train prior networks. Wu et al. [21] designed a deblurring method based on a two-stage wavelet-based convolutional neural network, which embeds discrete wavelet transform to separate image context and texture information, and reduces computational complexity. In addition, they modified the initial module by increasing the pixel attention mechanism and the channel scale factor so that the weight of each convolution kernel was changed, and at the same time, the receiving field was increased and the parameters of the module were significantly reduced. In order to guide the network to perform higher-quality deblurring and improve the feature similarity between the restored image and the clear image, SharpGAN [22] proposes a method that combines feature loss of different levels of image features. In addition, they introduced the network into the receiving domain block network to improve the ability to extract fuzzy image features. Wang et al. [23] proposed a new framework that uses depth variational Bayes to blindly deblur the image. This framework uses discrete reasoning and deep neural networks (DNNs) to jointly estimate the posterior of potential clean images and blur kernels. In addition, under the guidance of the lower bound of evidence, the data of clean images and blur kernels can be considered. Drive a priori supervision and physical fuzzy models to train the inference DNNs involved. MPRNet [24] proposed a method, which has two characteristics. One is that information is exchanged in the order from early to late; the other is to avoid information loss. It is also in the feature processing block. A horizontal connection is added, and a tightly connected multi-level architecture is created on this basis.

## 3. Proposed Method

### 3.1. Overview

**Figure 1:**
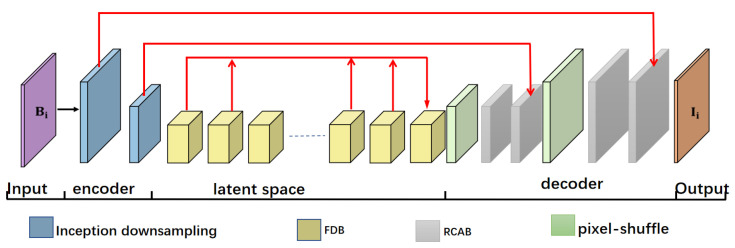
Overview the frequency disentanglement distillation image deblurring network (FDDN).

**Figure 2:**
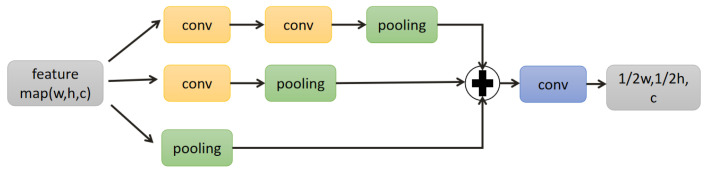
Inception down-sampling block.

**Figure 3:**
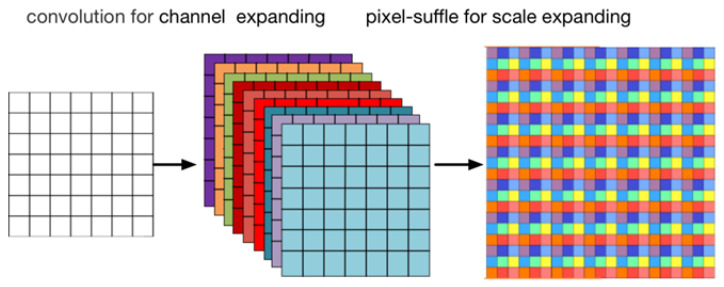
The architecture of pixel-shuffle block.

In this article, we also constructed an encoder–decoder network structure as shown in Figure 1, and it can be divided into three parts. In the first part, the structure of the encoder is mainly composed of two identical inception down-sampling blocks, as shown in Figure 2. The inception down-sampling is inspired by the inceptions block [25], where the down-sampling operation has been completed by max-pooling with a kernel of 2 × 2 and a stride of 1, so that the length and width of the feature map are each 1/2 of the original length and width. Before pooling, the feature is resampled with the convolution kernel 3 × 3 and the stride is 1 without changing the size of the feature map. The receptive field of the feature pixel will expand due to the resampling, thus solving the problem of detail loss in the process of feature map scale reduction. After the encoding path, it enters the second part. The middle layer feature in the latent space completes the operation of frequency disentangle and distillation here through 16 frequency distillation blocks(FDB) to reduce the disturbance of the network by useless features. We will introduce it in detail in Section 3.2. The third part is the decoder, in which the gray module is the residual channel attention block [26], which is also used for feature reorganization, also does not change the size of the input feature map, but through its channel attention mechanism to make the decoding network more targeted recovery Image details. The green module is pixel-shuffle convolution [27], which uses convolution to expand the number of feature channels without changing the size of the feature map. Then squeeze the feature maps of multiple channels into one feature map to achieve the purpose of up-sampling, as shown in Figure 3. Since the expanded feature channels are obtained by convolution layers, these convolutions can be trained together with other parameters, so compared with the traditional interpolation up-sampling method [16,17], it can produce more realistic results. Furthermore, in our frequency disentangle distillation image deblurring network (FDDN), we design a large number of skip connection mechanisms [17] between encoding path and decoding path.

### 3.2. The Algorithm Frequency Split Block

**Figure 4:**
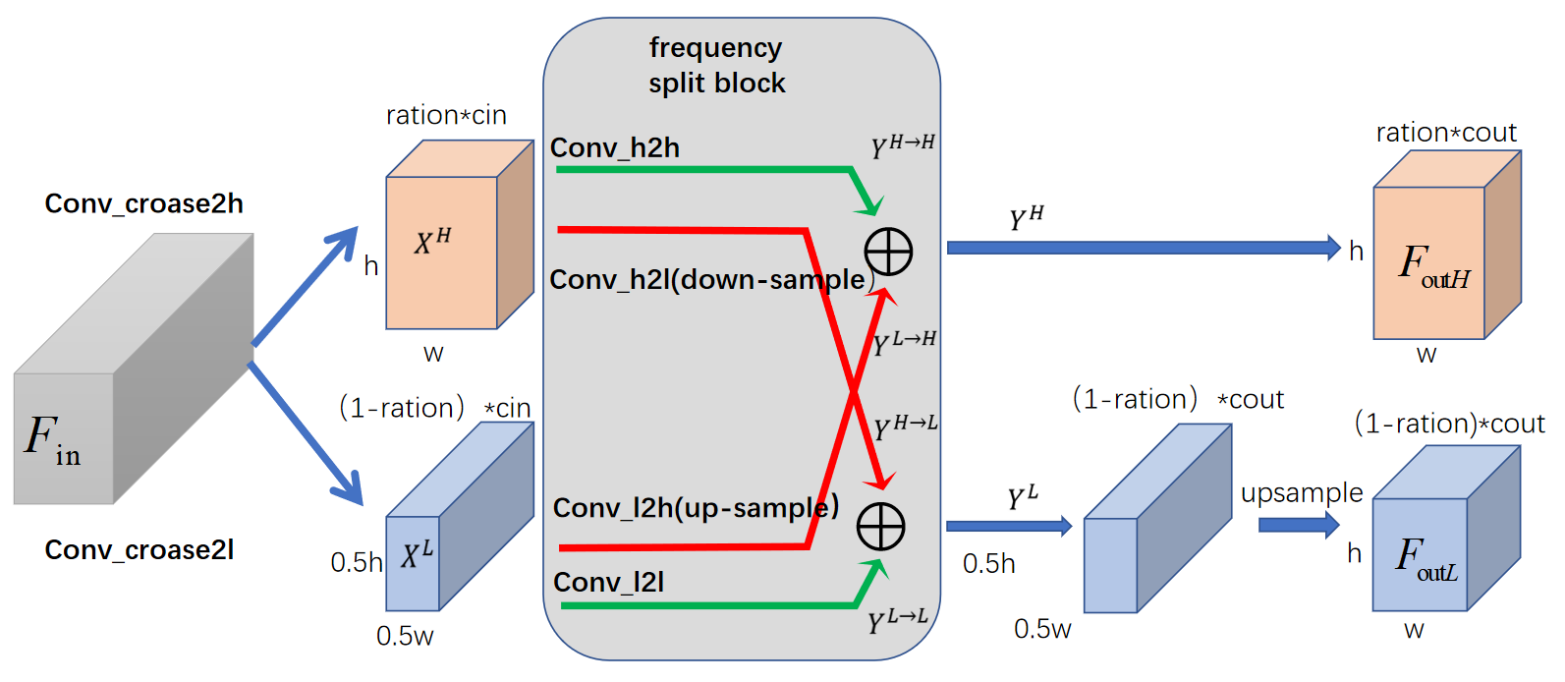
The construction of frequency split block(FSB). The gray block represent input feature maps of FSB. The blue represents low frequency channel and the red means high frequency channels.

Inspired by octconv [15], and in response to the needs of our network itself, we propose a frequency-based split module. As shown in Figure 4, Where the grey block representing the input feature map, red block representing high-frequency feature map, and blue block representing low-frequency. The frequency split block(FSB) is the component of the frequency distillation block. It is responsible for completing the channel split task during the distillation. Through the channel split, the useful part is retained, and the less useful part continues to be recursively distilled. Frequency split block splits the channels from the perspective of high and low frequencies, making the network more interpretable and efficient. The frequency split block still follows the design of octconv [15], which can carry out communication in intra-frequency, as well as inter-frequency. FSB is roughly divided into the following four steps, as shown in Table 1. Step 1, we determine the hyperparameter ratio. In order to meet the flexibility of the network, we can set different ratios according to the distribution of the dataset. The high frequency channel number is HChannel, and the low frequency channel number is LChannel. In the Step 2, the input feature map through Conv_croase2h and Conv_croase2l divided into two parts, feature to high and feature to low. This time, the high and low frequencies that are distinguished are only roughly divided. In the subsequent of the algorithm, the intra-frequency and the inter-frequency communication are are distinguished in more detail. Since the mostly redundant information in low-frequency, we perform down-sampling in the low-frequency information. Here, the down-sampling is done by a convolution operation with the stride of 2. The Step 3 is to complete the intra-frequency communication by convh2h and convl2l. The Step 4 is inter-frequency communication. In the conversion process between high and low frequency, it will be accompanied by the transformation of the feature scale. In order to remove the redundancy of low-frequency information. Finally, through such a frequency dimension-based disentanglement method, the input features are generated under the operation of keeping the size unchanged, and two different features of high and low are generated. The parameters of convolution are shown in Table 2.

### 3.3. Frequency Distillation Block (FDB)

**Figure 5:**
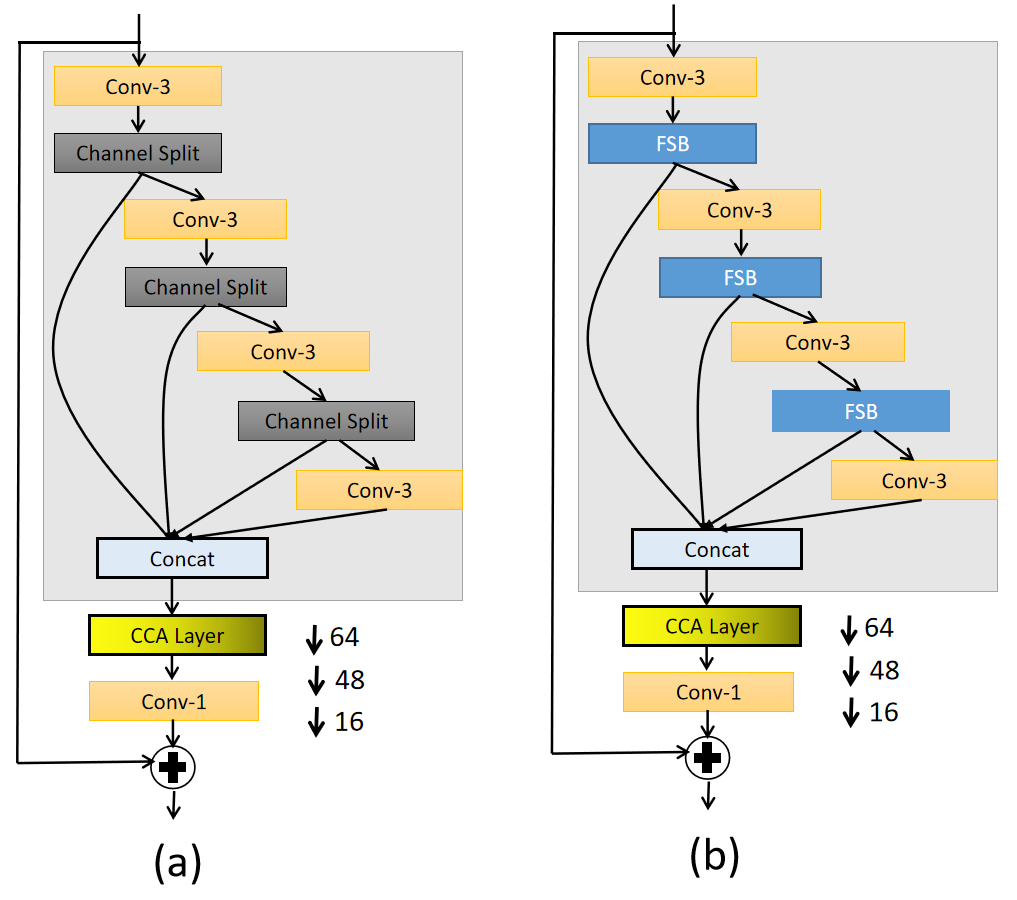
(**a**) represent tradition distillation block; (**b**) the disgn of FDB.

Frequency distillation block is used in latent space. There are two reasons for this. The first is to improve the performance of the network. There are high-dimensional features in the latent space, and the feature size is generally small, which makes the amount of calculation less during the convolution operation. Second, our purpose is to distinguish feature information in the frequency dimension. High-dimensional features make it easier to distinguish between foreground semantic information and background information, as well as areas with rich features and flat gradients. This makes the high-dimensional features in latent space suitable for entangled operations. As show in Figure 5a, The traditional distillation block [28] is a progressive refinement module. It employs three channel split operations on the preceding features, which will produce two-part features. This one will be sent directly through the jump link mechanism in the feature fusion stage; this part’s channels are regarded as the useful information for restoring the image. The remaining part will be sent to the next recursive channel split operation. However, it simply divides the feature channel only according to a ratio. The detail of traditional distillation block [28] is show in Equations (2)–(5).

(2) $$F_{distilled1},\;F_{coarse1}=Split_{1}\left(L_{1}\left(F_{in}\right)\right)$$

(3) $$F_{distilled2},\;F_{coarse2}=Split_{2}\left(L_{2}\left(F_{coarse1}\right)\right)$$

(4) $$F_{distilled3},\;F_{coarse3}=Split_{3}\left(L_{3}\left(F_{coarse2}\right)\right)$$

(5) $$F_{distilled4}=L_{4}\left(F_{coarse3}\right))$$

Therefore, in this article, we propose a frequency distillation block (FDB), as show in Figure 5b, which is regarded as a frequency-based disentanglement. FDB uses the frequency split block (FSB) of Section 3.1 mentioned above. *F*${}_{low_{n}}$(*n* = 1, 2, 3) is the low-frequency feature in the feature map, and *F*${}_{high_{n}}$(*n* = 1, 2, 3, 4) is the high-frequency feature. By keeping the high-frequency features directly, we think that the high-frequency features contain rich details such as the contours and edges of the foreground in the image. In addition, *F*${}_{low_{n}}$, which is sent to the recursive distillation process again, is the low-frequency feature. We believe that in the low-frequency feature, the gradient changes slowly. The low-frequency mainly contains information such as the color, lighting, and blur features of the image. Of course, it does not completely rule out the existence of some useful details, so through the next distillation, purification is retained. The operation after this is to complete the operation of FDB through feature fusion with the saved *F*${}_{high_{n}}$(*n* = 1, 2, 3, 4). Subsequent operations merge the saved *F*${}_{high_{n}}$(*n* = 1, 2, 3, 4) to get the output feature *F*${}_{out}$. The detail of FDB is show in Equations (6)–(11).

(6) $$F_{high1},\;F_{low1}=FSB_{1}\left(L_{1}\left(F_{in}\right)\right)$$

(7) $$F_{high2},\;F_{low2}=FSB_{2}\left(L_{2}\left(F_{low1}\right)\right)$$

(8) $$F_{high3},\;F_{low3}=FSB_{3}\left(L_{3}\left(F_{low2}\right)\right)$$

(9) $$F_{high4}=L_{4}\left(F_{low3}\right)$$

(10) $$F=CAT(F_{high4},F_{high2},F_{high3},F_{high4})$$

(11) $$F_{out}=CCA\left(F\right)$$

### 3.4. Loss Function

#### 3.4.1. Mse Loss

When training the generative adversarial network, it is necessary to compare the reconstructed image with ground truth by appropriate measurement. Usually, people use a pixel-by-pixel comparison loss function to measure the difference between the reconstructed image and the ground truth. However, using the pixel-by-pixel comparison loss function alone will produce artifacts. For example, consider two identical images that are offset from each other by one pixel; although they are very similar in perception, the results will be very different. In this case, the network will use the average of all possible solutions as the convergence value, which will cause artifacts. However, the pixel-by-pixel loss can still retain the detailed information of the picture to a certain extent. Therefore, we choose MSE as the pixel-by-pixel loss function, but we give it a relatively small weight. See Equation (12) for specific details.

(12) $$L_{MSE}=\frac{1}{wh}\sum_{x=1}^{w}\sum_{y=1}^{h}{\left({\left(l_{i}\right)}_{x,y}-G_{\theta G}{\left(B_{i}\right)}_{x,y}\right)}^{2}$$

Among them, $B_{i}$ represents the input blurred picture, $G_{\theta G}$ represents the generation network, and $l_{i}$ is the standard clear image. The w and h are the length and width of the input/output image, respectively.

#### 3.4.2. Perception Loss

At the same time, research [29] shows that perception loss can make the generation network improve the image quality. It maps the real picture and the generated picture to the feature map of the deep network and then calculates the least square method based on the feature map. This solves the disadvantage of pixel-by-pixel loss and performs pixel-by-pixel difference on the mapped feature map so that even if there is a certain degree of displacement, it will not have much impact. The process is shown in Equation (13), where *w*, *h* is the length and width of the feature map, and the parameters of the feature map are obtained by the VGG-16 network in the ReLU 3_3 layer.

(13) $$L_{percep}=\frac{1}{wh}\sum_{x=1}^{w}\sum_{y=1}^{h}{\left(\varphi {\left(l_{i}\right)}_{x,y}-\varphi {\left(G_{\theta G}\left(B_{i}\right)\right)}_{x,y}\right)}^{2}$$

The total loss function is shown in Equation (14).

(14) $$L_{Total}=L_{MSE}+\lambda L_{Percep}$$

## 4. Experiments

### 4.1. Dataset

In order to prove the effectiveness of the frequency disentangle distillation image deblurring network (FDDN) more convincingly, and to avoid the situation that the network has excellent performance only on a specific dataset due to over-fitting. We will conduct comparative experiments on three different datasets.

GoPro [2] dataset uses GoPro Hero 4 camera to capture video sequences at 240 frames per second (fps). This dataset consists of 3214 pairs of blurry and sharp images with a resolution of 1280 × 720. Among them, 1111 pairs are used as the testset. Different from using the blur kernel to convolve on a sharp image to obtain a blurred image, GoPro [2] follows the approximate camera imaging process during the image generation process in the blur and integrates consecutive frames within a certain exposure time to highlight the exposure time. The movement of the object inside is caused by the artifacts caused by the displacement, thereby generating a blurred image, rather than assuming a specific movement and designing a complex blur kernel. Therefore, there are only pairs of sharp/blurred image pairs in the dataset, and with no blur kernel. This kind of deblurring dataset without kernel estimation, compared with the traditional synthetic deblurring dataset with uniform blur kernel, is in the foreground, and the static background shows more realistic spatial blur changes.

HIDE [30] dataset is carefully constructed for human-aware image deblurring, covering a wide range of scenes, motions. HIDE dataset has 8422 sharp and blurry image pairs, extensively annotated with 65,784 human bounding boxes. For evaluation purposes, the images are split into separate training and test sets. Following random selection, we arrive at a unique split containing 6397 training and 2025 test images.

In this paper, we set a new Karate dataset in real scenes. It is difficult to get a sharp image completely corresponding to the blurred image after obtaining the blurred image. Even if certain conditions are deliberately created, slight deviations are unavoidable. Therefore, most of the benchmark datasets are obtained by the synthesis to obtain the paired images at this stage. There is no way to verify the ability of the algorithm to deblur the blurred image directly obtained in the real world. Therefore, we built a blurted dataset of the real scene. The main scene of the dataset is a karate match, and it is unpaired, with only blurred images and no corresponding ground truth.

### 4.2. Training Details

**Figure 6:**
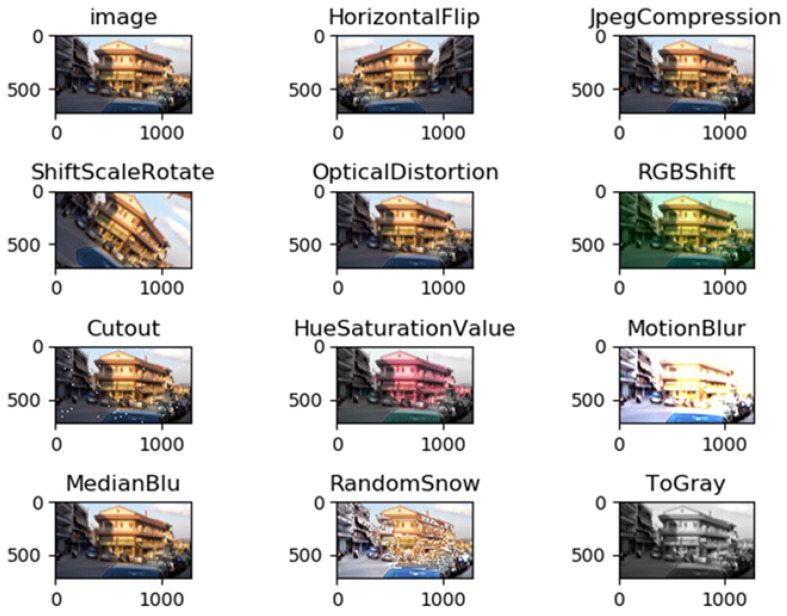
The visual represent of data enhancement.

The experience environment parameters were as follows: Intel Core i5 9400F CPU@2.9GHz; memory: 32.00 GB; operating system: Ubuntu18.04; GPU: Nvidia RTX2080Ti. We obtained the following fixed parameters through repeated experiments and adjustments: ratio = 0.5; λ = 0.1. The training uses 2500 epochs. The learning rate is 0.001, and each iteration attenuates 0.0000001 after 500 epochs; the optimizer uses adam; it was trained on a RTX2080Ti for about 14 days. Since it is a fully convolutional network, images of any size can be accepted. Data enhancement options such as horizontal flip, quality compression, rotation, optical transformation, color change, cropping, hue saturation transformation, motion blur, median blur, snow scene and grayscale image conversion, are shown in Figure 6.

### 4.3. Quantitative and Qualitative Evaluation on Gopro Dataset

**Figure 7:**
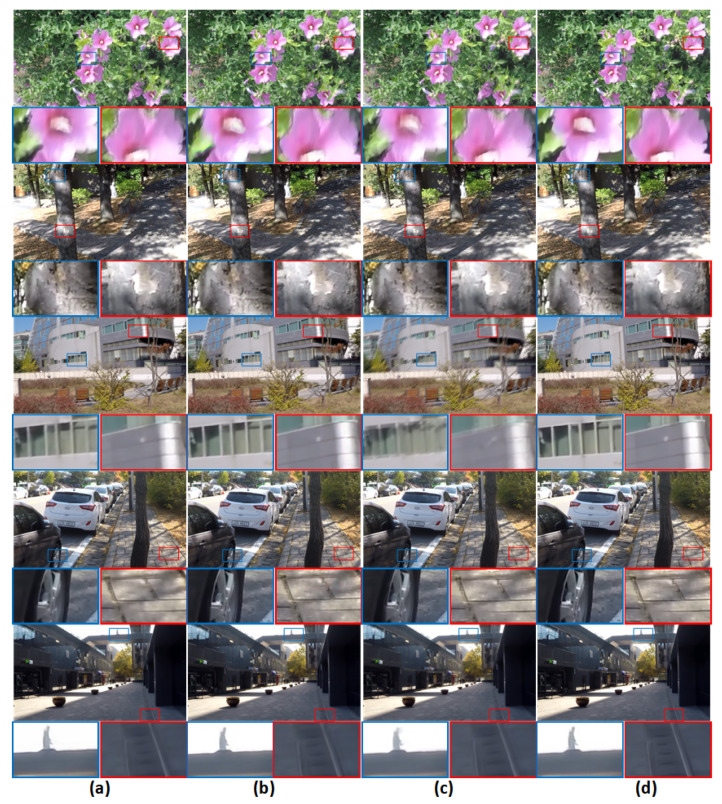
Visual comparison example on the GoPro dataset. (**a**) DMPHN [16]; (**b**) Gao et al. [17]; (**c**) Tao et al. [31]; (**d**) Ours.

We evaluate the performance and efficiency of our model in the GoPro [2] dataset. We make comparisons with the state-of-the-art deblurring methods [2,16,17,27,28,29] in Pre-Processing, in terms of PSNR, SSIM, model size and inference time for images. The quantitative results are shown in Table 3. Visual comparisons are shown in Figure 7.

### 4.4. Quantitative and Qualitative Evaluation on Hide Dataset

**Figure 8:**
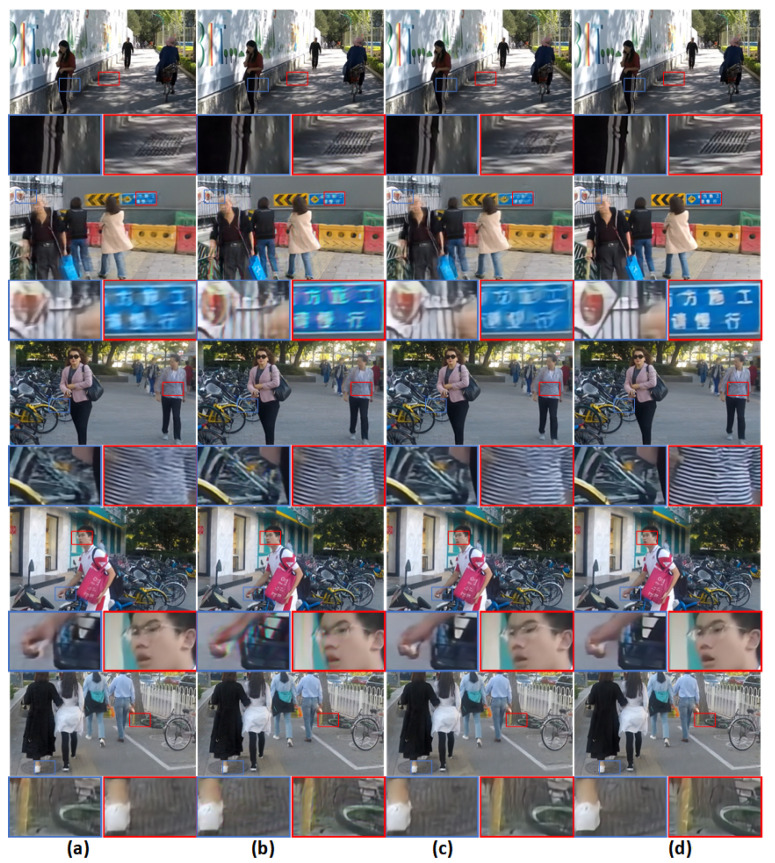
Visual comparison example on the HIDE dataset. (**a**) DMPHN [16]; (**b**) Tao et al. [31]; (**c**) Kupyn et al. [5]; (**d**) Ours.

To verify the validity of our method, we further evaluate our approach on the HIDE testing set [28]. In Table 4, we show a comparison with some of the methods. Visual comparisons are shown in Figure 8. From the data, we can see that our proposed method performs very well on this database.

### 4.5. Qualitative Evaluation of the Real-World Dataset

**Figure 9:**
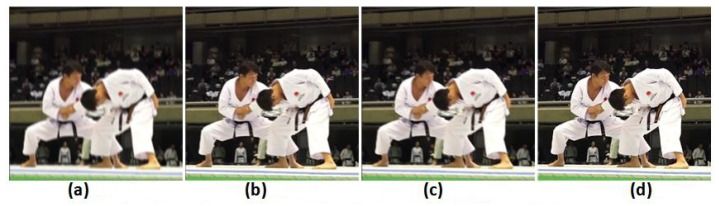
Visual comparison example on the karate dataset. (**a**) DeepDeblur [2]; (**b**) Deblur [5]; (**c**) Zhang et al. [3]; (**d**) Ours.

In this section, we have collected a set of datasets about karate competitions. The dataset is unpaired, with only blurred images and no ground. Therefore, PSNR and SSIM cannot be calculated without ground truth. Only in Figure 9 is the deblurring effect visualized.

### 4.6. Ablation Study

In this part, we verify the effectiveness of the method proposed in this paper through 3 sets of comparative experiments. The verified modules are frequency split block, distillation block, and frequency distillation block. In the first set of ablation experiments, we replaced the distillation block with resnet [35]. In order to preserve the frequency split block, we added the frequency split block to the feature pixel level to achieve feature fusion. We found that the absence of the distillation block will also greatly affect the model effect. In the second set of ablation experiments, to eliminate the frequency split block, we used RFDB [27] to replace the frequency distillation block. The channel segmentation of RFDB [27] uses a single convolution operation. Because a simple structure is used to replace a complex structure, there will be a slight advantage in the model size. However, through experimental results, it is found that the deblurring effect of the model will be affected. In the third set of ablation experiments, we used resnet [35] instead of the complete frequency distillation block. It was found that the deblurring ability of the model dropped the most in all ablation experiments, indicating that no matter the distillation block, the frequency split block, or the frequency distillation block composed of them, they all played a key role in the model. The specific quantitative data can be seen in Table 5.

### 4.7. Analysis of the FDDN

**Practical advantages:** FDDN has achieved convincing results in the three parameters of PSNR, SSIM and model size, which means that this model has certain advantages in running speed and running effect. Due to the design of the FDB, the FDDN has a very deep model structure, which means every pixel of feature map have a very large receptive field. This property allows the image restoration process can make better use of the surrounding pixel information to restore the image details. The details can be see in Figure 8. In addition, according to Experiment 4.6, it can be seen that FDDN can not only recover image blur well on public datasets, but also generalize to specific application scenarios, such as motion blur in karate.

**Disadvantages:** FDDN cannot directly solve the entanglement of the blur and content information, but indirectly realizes the entanglement of blur information and content information through distillation operation in the two dimensions of high and low frequency. High-frequency information is retained as far as possible, and the redundancy of low-frequency information is eliminated.

## 5. Conclusions

Image deblurring is an important technical means to ensure the quality of the image. In this paper, we hope to realize the disentanglement of blur information and content information from the perspective of frequency. Therefore, we proposed the frequency disentanglement distillation image deblurring network (FDDN), which have three contribution: first, we proposed the frequency split block (FSB), which can distill high-frequency and low-frequency in defferent channels. Second, frequency distillation Block (FDB), which is a combination of frequency split block (FSB) and distillation block. FDB can be regarded as a frequency-based disentanglement. By keeping the high-frequency features directly and sending the low-frequency feature to the recursive distillation process, FDB distillate the useful feature step by step. Third, we perform extensive experiments on the tasks of motion deblurring using both synthetic datasets and real images and achieve an efficient result. We find that the FDDN have a good ability of generalization, and it can restore the details of blurry area effectively.

In the following work, we will further explore and improve the image restore ability of FDDN, which is not only used for image blurring, but also can be extended to image derain, super resolution, image inpainting and other joint tasks. In addition, the transformer mechanism will be introduced to further improve the quality of image restore. Finally, we will reduce the parameters of the model, so that FDDN can complete the real-time deblurring task.

## Figures and Tables

**Table 1:** The algorithm frequency split block.

**Input:** $F_{in}\left(w,h,input\right),ratio,input\_channel,output\_channel$
**Step1:** Hchannel = ratio * output_channnel
Lchannel = (1-ratio) * output_channel
**Step2:** Feature to High = lucky_relu[Conv_croase2h(Feature in)]
Feature to Low = lucky_relu[Conv_croase2l(down-sampling(Feature in))]
**Step3:** H2h = Conv_h2h(feature to high)
L2l = Conv_l2l(feature to low))]
**Step4:** h2l = lucky_relu[Conv_h2l(down-sampling(feature to high))]
L2h = lucky_relu[Conv_l2h(up-sampling(feature to low))]
**Output:** $F_{h}=H2h+L2h$
$F_{l}=up-sampling\left(L2l+h2l\right)$

**Table 2:** The detail parameters of the convolution layer of FSB.

Conv_Name	Input_Channel	Output_Channel	Kernal-Size	Stride
Conv_croase2h	input_channel	Hchannel	3	1
Conv_croase2l	input_channel	Lchannel	3	1
Conv_h2h	Hchannel	Hchannel	1	1
Conv_h2l	Lchannel	Lchannel	1	1
Conv_l2l	Lchannel	Lchannel	1	1
Conv_l2h	Lchannel	Hchannel	1	1

**Table 3:** Performance and efficiency comparison on the GoPro dataset.

Methods	PSNR	SSIM	Model Size (MB)	Time (s)
DeepDeblur [2]	29.08	0.841	303.6	15
Zhang et al. [3]	29.19	0.9306	37.1	1.4
Gao et al. [17]	30.92	0.9421	2.84	1.6
DeblurGAN [5]	28.70	0.927	37.1	0.85
Tao et al. [31]	30.10	0.9323	33.6	1.6
DeblurGANv2 [9]	29.55	0.934	15	0.35
DMPHN [16]	30.21	0.9345	21.7	0.03
SIS [32]	30.28	0.912	36.54	0.303
Yuan et al. [33]	29.81	0.936	3.1	0.01
Pan et al. [20]	31.40	0.947	-	-
Wu et al. [21]	30.75	0.913	29.1	3.2
SharpGAN. [22]	29.62	0.897	-	0.17
Ours	**31.42**	**0.923**	**8.08**	**0.019**

**Table 4:** Performance comparison on the HIDE dataset.

Methods	Sun et al. [34]	DMPHN [16]	Nah et al. [2]	Tao et al. [31]	Kupyn et al. [5]	GCResNet [19]	FDDN (Ours)
PSNR	23.21	29.09	27.43	28.60	26.44	30.04	**30.07**
SSIM	0.797	0.930	0.902	0.928	0.890	0.924	**0.923**

**Table 5:** Quantitative comparison of different ablations of our network on the GroPro dataset.

Distillation Block	Frequency Split Block	Frequency Distillation Block	PSNR	SSIM	Model Size (MB)
✕	✓	✓	29.65	0.892	9.05
✓	✕	✓	29.80	0.901	7.96
✓	✓	✕	**29.21**	**0.863**	**7.89**
✓	✓	✓	**31.42**	**0.923**	**8.08**

## Data Availability

The code of FDDN can be available at https://github.com/yimingliu123/FDDN (accessed on 7 June 2021).
